# The rising global burden of stroke

**DOI:** 10.1016/j.eclinm.2023.102028

**Published:** 2023-05-23

**Authors:** eClinicalMedicine

May marks annual stroke awareness month in the UK and USA. According to the most recent Global Burden of Disease (GBD) estimates, there were around 12.2 million incident cases of stroke, 143 million disability-adjusted life-years (DALYs) lost, and 6.6 million deaths globally in 2019, making stroke the second leading cause of death and third leading cause of disability worldwide. Over the past 30 years, there has been an increase in the absolute number of incident (70%) and prevalent (85%) strokes, as well as deaths (43%) from stroke. In addition to population growth and ageing as contributors to this rise in global stroke burden, it is thought that increased exposure to five leading risk factors (systolic blood pressure, high body-mass index, high fasting plasma glucose, ambient particulate matter pollution, and smoking) has also played an important role. The total global direct (eg, treatment, rehabilitation, and social care) and indirect costs (eg, income losses) of stroke in 2017 were estimated to be US$891 billion, equivalent to 1.12% of global GDP. The large and continued rise in the global burden of stroke presents many challenges to governments and health-care systems worldwide that need to be urgently addressed.

Stroke is caused by impaired perfusion through the blood vessels to the brain. The most common type is ischaemic (thrombotic or embolic) stroke, followed by haemorrhagic (intracerebral or subarachnoid) stroke. Other types include transient ischaemic attacks (caused by a serious temporary clot) and cryptogenic strokes (of unknown cause). In addition to modifiable risk factors, non-modifiable risk factors such as age, sex, family history, and genetic factors can also play a role. Although stroke-related disability rates are higher in men, the incidence and prevalence of stroke is higher in women. Symptoms vary widely, but the most common include weakness of the face, arm, or legs typically on one side of the body, problems with comprehension, and severe headaches. Diagnosis involves measuring blood flow to the brain or brain imaging by CT, MRI, CT or magnetic resonance angiography, or Doppler sonography. Restoring blood flow to the brain and treating neurological damage are the primary aims of stroke treatment, which can include intravenous or intra-arterial thrombolytics or fibrinolytics, craniotomy, or mechanical thrombectomy. The benefits of therapy are time-dependent, and treatment should be administered as quickly as possible. Improvements in acute stroke care have led to a greater proportion of people surviving the acute episode. However, many survivors are left with short or long-term disabilities that markedly affect their quality of life, such as impaired cognitive function, psychological issues, or physical impairments. Rehabilitation can involve a combination of physical, occupational, speech, or cognitive therapies, and psychological support to facilitate functional independence. Given that type and severity of impairments vary widely between patients, there is no universal treatment for the consequences of stroke, making management of these patients particularly challenging. Rapid diagnosis and prompt delivery of treatment are crucial factors in determining outcomes, as delays in reperfusion can increase the risk of death, level of disability, and the long-term effects of stroke.

There has been considerable interest in emerging neuroprotective therapies and the use of artificial intelligence (AI) to improve stroke care, particularly with regards to rehabilitation. For instance, the first-in-class Toll-like receptor 4 antagonist, ApTOLL, has shown promise in reducing ischaemic damage in preclinical models, and its safety and biological effects are being tested in a human phase 1b/2a clinical trial. In addition, a novel vagus nerve stimulation system combined with rehabilitation was found to provide clinically meaningful changes in impairment versus sham stimulation in patients with long-term moderate-to-severe arm impairment after ischaemic stroke. Similarly, the Brainomix e-Stroke system, developed in the UK, uses AI algorithms to provide decision support to clinicians in the interpretation of brain scans, potentially improving the detection of ischaemic changes.

While acknowledging these advances, it is important to also consider the substantial and ever widening geographic and economic disparities in stroke burden worldwide. Almost 90% of all deaths and disability from stroke occur in lower income and lower-middle-income countries, particularly in sub-Saharan Africa and Asia. Estimates also suggest that age-standardised mortality and disability rates were almost four times higher in the World Bank low-income group than in the high-income group in 2019. Furthermore, over 80% of individuals in low-income countries (LICs) die within 3 years of a stroke compared with around 34% in high-income countries (HICs), such as Sweden.

A World Stroke Organization-WHO survey investigating the status of stroke services in 84 countries found that only 18% of LICs have stroke units (*vs* 91% of HICs) and only 26% of LICs offered acute treatment (*vs* around 60% of HICs). Clinicians in low-resource settings also face barriers in access to medical imaging devices, making it difficult to distinguish between the types of stroke and deliver appropriate care. Furthermore, awareness of stroke in the general population could be lower in LICs, and there is substantial variation in the prevalence and importance of individual risk factors for stroke between LICs and HICs. The lack of stroke awareness, and limited access to stroke units and brain imaging contribute to delays in access to appropriate care, increasing the risk of poor outcomes.

The increasing global burden of stroke indicates that more effective strategies for the prevention and management of this disease are needed. While research into novel therapies and strategies to improve existing stroke care are critical, the vastly unequal distribution in global stroke burden necessitates future public health and research efforts to focus on closing the existing gaps between low-resource and high-resource settings by developing and implementing evidence-based, targeted stroke prevention policies and management strategies that enable access to timely, effective care.

